# Spatiotemporal evolution and driving mechanism of regional shrinkage at the county scale: The three provinces in northeastern China

**DOI:** 10.1371/journal.pone.0271909

**Published:** 2022-08-22

**Authors:** Shangkun Yu, Chengxin Wang, Zhenxing Jin, Shuai Zhang, Yi Miao

**Affiliations:** 1 College of Geography and Environment, Shandong Normal University, Jinan, China; 2 Collaborative Innovation Center of Human‑Nature and Green Development in Universities of Shandong, Shandong Normal University, Jinan, China; 3 Communist Party China, Shandong Provincial Party School, Jinan, China; Tohoku University, JAPAN

## Abstract

The three northeast provinces are typical areas of regional shrinkage in China. A scientific understanding of their shrinkage and driving mechanism is conducive to the transformation and development of traditional industrial bases in China. This study analyzed the spatiotemporal evolution and driving mechanism of regional shrinkage at the county scale in the three provinces. The main findings are as follows: (1) 40.86% of counties in the three provinces shrank, forming three concentrated shrinking regions. However, comprehensively shrinking regions were narrowed and lessened with the introduction of the Northeast Area Revitalization Plan. (2) The population-related shrinking regions accounted for more than 90% and continued to expand. Such shrinkage was higher in the north than in the south. The degree of economy-related shrinkage was the most serious, and the hotspots were mainly concentrated in Liaoning Province. The scope of space-related shrinkage was most minor, and such shrinkage was relatively mild. (3) When it came to influencing factors, the shrinkage index was positively correlated with the proportion of the secondary industry, the output value of agriculture, forestry, animal husbandry and fishery, the number of industrial enterprises above the designated size, fiscal expenditure, and the balance of resident deposits, and negatively correlated with the altitude, the proportion of the tertiary industry, and population aging. Geographically weighted regression (GWR) and ordinary least squares (OLS) produced similar regression results. The spatial pattern of influencing factors was consistent with the hotspot areas of population-related shrinkage or economy-related shrinkage, with significant spatial differences.

## Introduction

Shrinkage, as an objective stage in the process of regional growth and development [1], represents a huge challenge facing China in its new urbanization in the future [2]. The shrinkage of a city—the center of a region, has attracted much attention. Urban shrinkage first took place in developed countries in Europe and America. For example, there was a loss of urban population in the United States after World War II [3], and shrinkage of urban core areas also occurred in Germany in the 1960s and 1970s [4]. Depopulation was used as a measure of urban shrinkage in the early days. For example, according to the Shrinking City International Research Network, in a city with no less than 10,000, depopulation over two years represents urban shrinkage [5]. In terms of classification, the most typical shrinking cities are “perforated” cities and “pie-shaped” cities, represented by old industrial cities in Europe and the United States, respectively [6, 7]. With the rapid expansion of cities in China and the continuous accumulation of population in cities, local urban shrinkage is becoming increasingly apparent [8, 9]. It occurs not only in relatively slow-developing resource-based cities but also in cities in economically developed areas [10, 11].

Compared with urban shrinkage, regional shrinkage includes not only the shrinkage of physical urban areas or urbanized areas but also the shrinkage of large and small towns and rural areas with larger scales and broader scopes. Small towns and smaller villages are more susceptible to shrinkage due to the siphonic effect of surrounding cities because they are too small and have single functions [12, 13]. Under China’s current administrative division system, the research will satisfy the need of development planning in China if it can take cities, small towns, and villages as one research unit and explore the shrinkage of the entire region. Existing research in regional shrinkage focuses on the urban scale, while few studies are dedicated to rural areas [14, 15]. In terms of quantitative measurement, population change has been widely used as the core feature of regional shrinkage, measured by the reduction of the total regional population, population density, and negative population growth rate in a specific period [16, 17], followed by comprehensive identification of population, economy, society, and space [18]. In the identification of regional shrinkage, some studies have found that there are significant differences in the shrinkage pattern between China and other countries: European and American countries are characterized by hollow central cities and expanding suburbs; however, China is dominated by the expansion of central cities and the shrinkage of small marginal towns and villages [19, 20]. Existing research on regional shrinkage in China demonstrates the following characteristics: First, the discussion about theory and mechanism has become more mature. For example, the life cycle of city and economic cycle theories were employed to explain shrinkage [21], and experience from western countries was used to explore regional sustainability in shrinkage [22]. Secondly, the measurement indicators are becoming more abundant and appropriate. Demographic depression, as an important indicator of regional shrinkage, has been widely used in related studies [23]. The combination of population and economy has also become an important indicator for regional shrinkage [24, 25]. In addition, there are multi-dimensional indicators for shrinkage [26] and the regional development indicator system [18]. Finally, the source of data becomes more diverse, including not only statistical data from statistical yearbooks and demographic censuses but also remote sensing data [27]. Generally, the theoretical and empirical research on regional shrinkage is fruitful, albeit problems such as incomplete evaluation standards, insufficient research scale, and unclear research objects. Although it has been recognized that regional shrinkage is an evolutionary process in multiple dimensions, the existing evaluation standard focuses heavily on population changes, and there is a lack of specific definitions for each dimension in the few multi-dimensional measures of regional shrinkage. Most studies pay attention to the scale of prefectures and cities alone, but little attention to empirical research on grass-roots cities at the county level. Some studies even mistake the concepts of “city” and “region”, thus viewing “urban shrinkage” as “regional shrinkage”.

To fill the gap mentioned above, this paper comprehensively evaluates the phenomenon of regional shrinkage from the three dimensions of population, economy, and space, clarifies the conceptual connotation of regional shrinkage and the specific definitions of each dimension, and explores the relationship between different dimensions of shrinkage, thereby enriching the theoretical and evaluative system of regional shrinkage and deepening people’s understanding of regional shrinkage. The three northeastern provinces with the most severe and most typical shrinkage in China are selected as the research area [28, 29], and the county (county-level city, county, autonomous county) is the basic research unit. This study builds an evaluation indicator system to comprehensively measure the regional shrinkage of these provinces based on statistical data and night-time light datasets from the three dimensions of population, economy, and space. In so doing, it attempts to illuminate the overall pattern and driving mechanism of regional shrinkage in the three northeastern provinces. On this basis, it develops an indigenous research paradigm for regional shrinkage in China and provides more evidence for the international research community from China’s perspective. The present study pays attention to the regional shrinkage at the county scale, which is expected to reflect the spatiotemporal pattern of regional shrinkage in these provinces more properly, propose measures for formulating regional planning in these shrinking regions, and provide a reliable theoretical and empirical reference for the future spatial layout of this region.

## Study area and methods

### Study area

**Fig 1:**
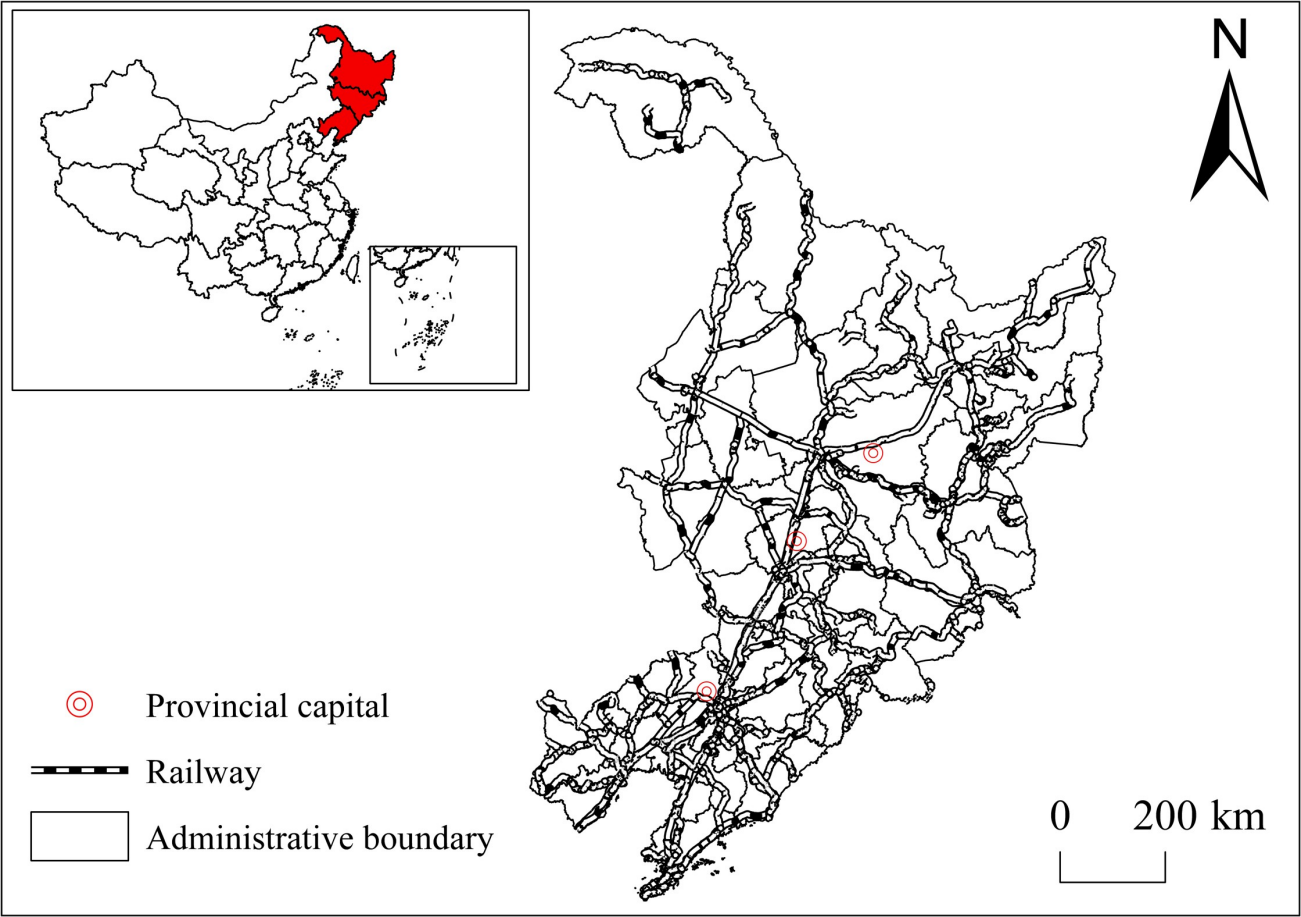
Administrative division and railways of the three northeastern provinces. Note: The basic map came from a public map from the standard map service website of the Ministry of Natural Resources of China. The drawing approval number is GS (2016)1593.

The three northeastern provinces refer to Heilongjiang, Jilin, and Liaoning provinces (Fig 1), with a total area of 787,300 km^2^. Jointly influenced by the “Northeast Phenomenon” in the 1980s and 1990s and the “New Northeast Phenomenon” since 2014, the three northeastern provinces with serious population loss have become typical areas for research on regional shrinkage in China. There are totally 34 prefecture-level and 281 county-level administrative divisions under the jurisdiction of the three provinces. We selected county-level administrative divisions for this study. Meanwhile, considering the commonality and indivisibility of the economic development and urban construction of the municipal district of a city, we regarded the municipal district of each city as a research unit. The administrative units involved in the adjustment of divisions were subject to the adjusted ones; as for the Yanbian Korean Autonomous Prefecture without a municipal district, the city of Yanji where the capital is located was considered as the municipal district, hence 177 research units in total.

Since the expansion and shrinkage of a region is periodic, this study explored the phenomenon in the period of 2012–2018. The year 2015 is a divide between China’s 12^th^ and 13^th^ Five-Year Plans. Since 2015, state leaders have attached greater importance to the revitalization of the old industrial base and the loss of population in Northeast China by their many visits to this region. Policy documents such as *Several Opinions of the CPC Central Committee and State Council on Comprehensively Revitalizing Old Industrial Bases in Northeast China* and *The 13*^*th*^ *Five-Year Plan for Revitalizing Northeast China* have laid a favorable foundation for the three northeastern provinces to cope with shrinkage and realize revitalization. Therefore, 2015 may be a turning point for them to address shrinkage. To explore whether shrinkage is a short-term phenomenon in regional development and reveal the evolution of regional shrinkage in the three northeastern provinces in more detail during the entire research period, this period was further divided into two shorter periods: 2012–2015 and 2015–2018. The data of municipal districts were mainly derived from the “China City Statistical Yearbook”; the data of county-level administrative divisions mainly came from the “Heilongjiang Statistical Yearbook”, “Jilin Statistical Yearbook”, “Liaoning Statistical Yearbook”, and “China County Statistical Yearbook” of the corresponding years; NPP-VIIRS night-time light datasets stemmed from the National Geophysical Data Center of the National Oceanic and Atmospheric Administration (NOAA) of the United States. The original NPP-VIIRS night-time light datasets were processed as follows: the data were converted into a Lambert equal-area projection coordinate system that is more suitable for the Chinese terrain, the NEAREST proximity method was used for resampling, and the county-level administrative division map of the three provinces was used as a mask to tailor the picture; the monthly light data of each year were combined into the annual data, and then the negative value, the unstable light source, noise, and extremely high values were eliminated. Afterward, the change in light radiation intensity at night was used to represent the change in space-related shrinkage.

### Concepts of regional shrinkage and the indicator system

Concepts such as “urban shrinkage” and “shrinking city” are imported to China from other countries. Since China and the international community have different definitions for the city, the research scope of these imported concepts is also different between China and other countries. According to the definition by the international community, spatial agglomeration of population and economic activities and density and compactness are the basic features of a city. By contrast, under the current administrative division system in China, a city can be defined both by the administrative region (the administrative scope of the city) and the physical region (the built-up area of the city and the scope of urbanization) [9, 30]. In most cases, the “city” still includes vast rural areas. Some researchers in China have misunderstood the concepts of “urban shrinkage” and “regional shrinkage”, or considered “regional shrinkage” as “urban shrinkage” in a broad sense. To this end, this paper distinguishes the two concepts and shifts its perspective from a “city” to a “region”, which can effectively overcome unclear boundaries of the study area and satisfy the needs of practical development under the administrative division of Chinese cities. Urban shrinkage refers to the shrinkage of the physical area or urbanized area of a city, mainly manifested in the municipal district or built-up area of a prefecture-level city and the streets of counties, while regional shrinkage is the shrinkage of the whole administrative division of the city, which reflects the overall shrinkage of different administrative units such as cities, towns, and villages. In short, the spatial scope of “urban shrinkage” is contained in “regional shrinkage”; in other words, the former is a “point”, while the latter is a “surface”. The two influence and interact with each other: on the one hand, the overall regional shrinkage caused by the loss of population leads to the loss of social capital and the reduction of public activities, resulting in a decline in the vitality of urban development [31]; on the other hand, the shrinkage of a city as the core of regional development weakens its role in driving the development of towns and rural areas, which in turn leads to the stagnation of the overall economic development and the outflow of the population in the region.

In terms of measurement indicators, the definition of regional shrinkage still refers to the identification method of urban shrinkage. The decrease in population size or population density is the main indicator of regional shrinkage [32], which has been widely used in indicating and identifying shrinkage, and its rationality has also been proven [33]. However, regional shrinkage is a comprehensive concept and cannot be characterized by population reduction alone [34], which otherwise can only be called regional population-related shrinkage, namely, regional shrinkage in a narrow sense. In a broad sense, regional shrinkage should cover many aspects such as population, economy, society, and space, and at the same time, conceptual differences and confusion of results caused by too many indicators should be avoided. Therefore, based on the three core dimensions of population, economy, and space, we define regional shrinkage as: the population loss characterizing the process of regional development, accompanied by economic recession or transformation, and shrinking space. Specifically, regional population-related shrinkage refers to population aging and declines in the total number and quality of employed population along with the continuous population loss in the region. Economy-related shrinkage refers to the industrial recession, government fiscal decline or even bankruptcy, and insufficient economic vitality due to low-end industrialization in the process of regional economic development. Space-related shrinkage refers to the decline and degradation of regional space [35], including the reduction of urban construction land and rural residential land, the weakening of land use intensity, and the emergence of “ghost towns” and “hollow villages”. which is manifested in vacant buildings and idle facilities. In this study, we built a regional shrinkage evaluation indicator system (Table 1) by integrating the three dimensions of population, economy, and space to comprehensively determine the shrinkage of the three provinces. The regional population was used as a measurement indicator, whose change can directly reflect the shrinkage in terms of population; economy-related shrinkage is an important manifestation of regional shrinkage where GDP can reflect the level of regional economic development, and public fiscal revenue can represent economic benefits; in the dimension of space, the area of urban construction land and rural residential land continues to increase due to the rigidity of space expansion. Night-time light datasets can reflect the vacancy of houses and the vitality of regional development, which has been applied in the measurement of regional shrinkage [36]. Therefore, night-time light datasets were selected to represent space-related shrinkage.

**Table 1:** Comprehensive evaluation indicator system for regional shrinkage of the three northeastern provinces.

Target layer	Criterion layer	Weight	Indicator layer	Weight
Regional shrinkage	Population	0.4	Regional population	1
	Economy	0.4	GDP	0.5338
			Government revenue	0.4662
	Space	0.2	Night-time light index	1

## Methods

### Shrinkage model

The shrinkage model was used to determine whether the area shrank. Taking population-related shrinkage as an example, it was calculated by the equation below:

$$S_{iP}=\frac{X_{iP}(2018)‐X_{iP}(2012)}{X_{iP}(2012)}$$

where *S*_*iP*_ refers to the population-related shrinkage index of area *i*; *X*_*iP*_ (2018) and *X*_*iP*_ (2012) refer to the numbers of the population of area *i* in 2018 and 2012, respectively. According to the calculation results, if the population-related shrinkage index *S*_*iP*_ is < 0, it indicates that the area has a shrinking population; otherwise, it has not a shrinking population. The measurement model of economy- and space-related shrinkage was consistent with population-related shrinkage, and the calculation of the shrinkage index in 2012–2015 and 2015–2018 was also consistent with population-related shrinkage. When *S*_*iP*_ < 0, the smaller *S*_*ip*_, the more severe regional shrinkage is. To objectively and reasonably describe the severity of regional shrinkage in the three northeastern provinces, it is necessary to conduct a standard classification of shrinkage. Based on the classification of the shrinkage index in the existing studies [37–39], and combined with the shrinkage index changes in the three northeastern provinces, the regional shrinkage of the study area was classified into severe shrinkage (S < -0.3), moderate shrinkage (-0.3 ≤ S < -0.1), mild shrinkage (-0.1 ≤ S < -0.05), and slight shrinkage (-0.05 ≤ S < 0).

### Subjective and objective weighting

The subjective and objective approaches were employed to assign values to the criterion layer and indicator layer. The weights of the criteria layer were determined by referring to the previous research results and consulting experts in related fields. As a highly mobile element in a region, the population is the core feature and the most direct manifestation of regional shrinkage; the economy is an important basis for the degree of regional development, which is sensitive to population loss and is a crucial feature of regional shrinkage. Therefore, the dimensions of population and economy should have greater weight. Night-time light datasets are an important indicator of the range and intensity of human activities, but they may have a lag in response to regional shrinkage, so the spatial dimension should have the smallest weight. The weight of the indicator layer was calculated by the entropy weight method. This method used to determine the weight of GDP total and fiscal revenue in the dimension of the economy can effectively avoid the randomness and presumption in the weight calculation process.

### Comprehensive evaluation with multiple indicators

The regional population-related, economy-related, and society-related shrinkage indexes of the three northeastern provinces were calculated by multiplying the standardized value of each indicator in the criterion layer and its weight and then summing them up. Then, the comprehensive shrinkage index of the region was produced by weighted summation using the following equation:

$$S_{c}=\sum_{i=1}^{n}r_{i}W_{i}$$

In the equation, *S*_*c*_ is the measurement index of the regional shrinkage criterion layer; *W*_*i*_ refers to the weight of the indicator; *n* represents the number of indicators included in the criterion layer; *r*_*i*_ is the quantized index value.


$$S=\sum_{j=1}^{m}{\left(S_{c}\right)}_{j}W_{j}$$

In the equation, *S* represents the regional shrinkage measurement index of the three northeastern provinces; (*S*_*c*_)_*j*_ refers to the measurement index of the population-related, economy-related, and society-related shrinkage; *m* is the number of criterion layers; *W*_*j*_ denotes the weight of the elements in criterion layers.

### Spatial autocorrelation

The global spatial autocorrelation model *Moran’s I* was employed to analyze the spatial agglomeration of the shrinking areas in the three provinces according to the following equation:

Moran'sI=n∑i=1n∑j=1nwij(xi−x¯)(xj−x¯)∑i=1n∑j=1nwij∑i=1n(xi−x¯)2,(i≠j)

In the equation, *n* represents the number of regions; *w*_*ij*_ is the weight of space; *x*_*i*_ and *x*_*j*_ are the shrinkage indexes of region *i* and region *j*, respectively. The value range of *Moran’s I* was [–1, 1]. A value > 0 means the shrinking area is in agglomeration spatially, and the larger the value, the greater the degree of agglomeration; a value < 0 indicates that the shrinking area is in dispersion, and the smaller the value, the greater the degree of dispersion.

### Ordinary least squares and geographically weighted regression

Ordinary least squares (OLS) is a global linear regression model, which can estimate global parameters for each influencing factor but does not consider spatial differences and their effects. Existing studies posit that if the independent variables have spatial autocorrelation, they cannot satisfy the null hypothesis that the residual items of the traditional regression model (OLS model) are independent. For this reason, geographically weighted regression (GWR), an extension of traditional regression analysis, is often used for supplementary research [40]. It includes sample location information to the regression parameters and reflects the non-stationarity of the parameters in space. Thus, this regression can be used to analyze the spatial heterogeneity of influencing factors in space. Since the diagnostic power of GWR is weak, OLS regression is usually required before GWR analysis to ensure the accuracy [41]. Therefore, in this study, OLS was used first to reflect the strength of each factor on the dependent variable, and then GWR was conducted to explore the spatial differentiation of the influencing factors. OLS and GWR were conducted by the equation below:

$$y_{i}=\beta_{0}(u_{i},v_{i})+\sum_{j=1}^{k}\beta_{j}(u_{i},v_{i})x_{\mathrm{ij}}+\varepsilon_{i}$$

In the equation, (*u*_*i*_, *v*_*i*_) is the center coordinate of the *i*^th^ sample space unit; *β*_*j*_ (*u*_*i*_, *v*_*i*_) is the regression coefficient of the *j*^th^ parameter of the *i*^th^ sample; *k* is the number of independent variables; *ε*_*i*_ represents the random error term.

## Results

### Spatiotemporal evolution of regional shrinkage in the three provinces

#### Hierarchical statistics of regional shrinkage in the three provinces

The analytic hierarchy process and entropy method were combined to calculate the weights of the criterion layer and indicator layer for regional shrinkage in the three provinces (Table 1). With reference to the previous research results and the suggestions of well-known experts in the field, the weight coefficients of population-, economy-, and space-related shrinkage were determined to be 0.4, 0.4, and 0.2, respectively. In terms of the indicator layer, the entropy method was used to determine the weights of the two indicators of the economic dimension, and a quantitative and hierarchical statistical analysis was performed on the 177 research units in the three provinces (Tables 2–4).

**Table 2:** Hierarchical statistics of regional shrinkage of the three northeastern provinces from 2012 to 2018.

	Severe shrinkage		Moderate shrinkage		Mild shrinkage		Slight shrinkage		Total	
	Quantity	Percentage (%)	Quantity	Percentage (%)	Quantity	Percentage (%)	Quantity	Percentage (%)	Quantity	Percentage (%)
Comprehensively shrinking region	5	2.82	47	26.55	9	5.08	11	6.21	72	40.68
Population-related shrinking region	0	0	16	9.04	66	37.29	78	44.07	160	90.4
Economy-related shrinking region	40	22.6	34	19.21	8	4.52	11	6.21	93	52.54
Space-related shrinking region	24	13.56	19	10.73	3	1.69	6	3.39	52	29.38

**Table 3:** Hierarchical statistics of regional shrinkage of the three northeastern provinces from 2012 to 2015.

	Severe shrinkage		Moderate shrinkage		Mild shrinkage		Slight shrinkage		Total	
	Quantity	Percentage (%)	Quantity	Percentage (%)	Quantity	Percentage (%)	Quantity	Percentage (%)	Quantity	Percentage (%)
Comprehensively shrinking region	0	0	42	23.73	25	14.12	18	10.17	85	48.02
Population-related shrinking region	0	0	4	2.26	22	12.43	121	68.36	147	83.05
Economy-related shrinking region	24	13.56	46	25.99	4	2.26	10	5.65	84	47.46
Space-related shrinking region	10	5.65	34	19.21	13	7.34	15	8.47	72	40.68

**Table 4:** Hierarchical statistics of regional shrinkage of the three northeastern provinces from 2012 to 2015.

	Severe shrinkage		Moderate shrinkage		Mild shrinkage		Slight shrinkage		Total	
	Quantity	Percentage (%)	Quantity	Percentage (%)	Quantity	Percentage (%)	Quantity	Percentage (%)	Quantity	Percentage (%)
Comprehensively shrinking region	0	0	16	9.04	25	14.12	30	16.95	71	40.11
Population-related shrinking region	0	0	5	2.82	21	11.86	140	79.1	166	93.79
Economy-related shrinking region	13	7.34	45	25.42	8	4.52	21	11.86	87	49.15
Space-related shrinking region	11	6.21	26	14.69	9	5.08	6	3.39	52	29.38

From 2012 to 2018, there were 72 comprehensively shrinking regions in the three provinces (Table 2), accounting for 40.68% of the total area, indicating the universality of regional shrinkage in the three provinces; the number of moderately shrinking regions was the largest, accounting for 26.55%. From the perspective of population, the number of population-shrinking regions accounted for as high as 90.40%, indicating the wide population loss during the process of urbanization in the three provinces; slight shrinkage was the most, accounting for nearly half. From the perspective of economy, there were 93 economically-shrinking regions, and most of them showed severe shrinkage, suggesting that although economy-related shrinkage was not as wide as population-related shrinkage, its degree was more serious. From the perspective of space, there were 52 spatially-shrinking regions, accounting for 29.38%. The number of severely, moderately, mildly, and sightly shrinking regions were 24, 19, 3, and 6, respectively. Due to its rigidity, space-related shrinkage was not as wide as population-related shrinkage, and its degree was not as severe as economy-related shrinkage. It was a dimension with relatively mild performance in the process of regional shrinkage in the three provinces.

Comparing the two periods (Tables 3 and 4), with the reduction in the scope and degree of comprehensive shrinkage, the number of economically shrinking regions was basically unchanged, but the degree lessened; the number of spatially-shrinking regions was reduced and the degree lessened; the degree of population-related shrinkage was relatively low, but the number of such regions was increasing. The number of comprehensively shrinking regions dropped from 85 in 2012–2015 to 71 in 2015–2018, indicating that some regions of the three provinces showed a trend of rejuvenation, which was inseparable from the country’s strategic support for the revitalization of northeast China. In terms of the degree of shrinkage, the number of comprehensively shrinking regions was reduced and the degree also lessened, with a decrease of 26 moderately shrinking regions. The number of regions with population-related shrinkage rose from 147 to 166. Although both periods were characterized by slight shrinkage, the scope expanded. The number of economically shrinking regions increased from 84 to 87, and the number was basically the same, but the degree of shrinkage declined slightly; the number of severely and moderately shrinking regions decreased by 11 and 1, respectively. The number of spatially shrinking regions plunged from 72 to 52. Except for an increase of 1 severely shrinking region, the number of moderately, mildly, and slightly shrinking regions showed a downward trend.

#### Spatiotemporal differences in regional shrinkage in the three provinces

**Fig 2:**
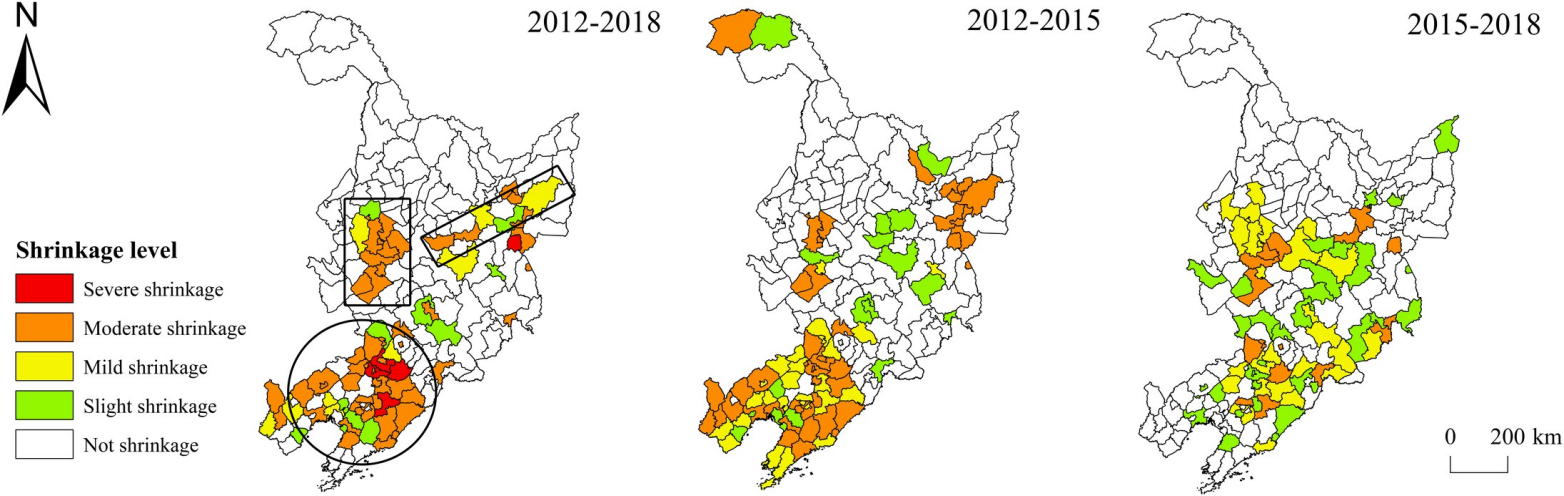
Spatial difference of comprehensive shrinkage of the three northeastern provinces. Note: The basic map came from a public map from the standard map service website of the Ministry of Natural Resources of China. The drawing approval number is GS (2016)1593.

Speaking of the spatiotemporal difference of the comprehensive shrinkage of the three provinces (Fig 2), there were three shrinking regions from 2012 to 2018: the “circular” region around the Shenyang municipal district, the “rectangular” region at the junction of Heilongjiang and Jilin provinces, and the “belt-shaped” region in the east of Heilongjiang province. 72.73% of the area in Liaoning province shrank, of which the “circular” region around the Shenyang municipal district was the largest. The main reason behind was that Shenyang, as the provincial capital and regional development center, had a strong siphon effect. The shrinkage range of Heilongjiang and Jilin provinces was relatively small. There was a moderately shrinking area at the junction of the two provinces, which mainly included the entire Daqing City and parts of Songyuan City and Suihua City. Daqing is a typical resource-exhausted city, whose population loss reached 67,000 in 2018; in the eastern part of Heilongjiang Province, there was a “strip-shaped” shrinking area across Harbin, Qitaihe, Jidong, and other cities. The resource-exhausted cities Qitaihe and Jixi’s municipal districts shrank, and the number of their population loss reached 101,000 and 75,000, respectively. The comprehensively shrinking regions of the three provinces from 2012 to 2015 were mainly concentrated in Liaoning Province, while the shrinking areas of Jilin and Heilongjiang provinces featured “dispersion at the macro level and agglomeration at the micro level”. In Liaoning Province, except for the municipal districts of Shenyang City and Chaoyang City, the other areas were mainly moderately and mildly shrinking. The mildly shrinking regions were distributed in a circular pattern around the municipal district of Shenyang City, while the moderately shrinking regions were mainly concentrated in its peripheral areas. From 2015 to 2018, the scope of shrinkage in Liaoning Province was significantly reduced, and the shrinking region was mainly distributed in the northern part of Liaoning Province and its junction with Jilin Province. In addition, there were also shrinking regions at the eastern edge of Jilin Province, the southern part of Heilongjiang Province, and the junction between Heilongjiang and Jilin provinces.

**Fig 3:**
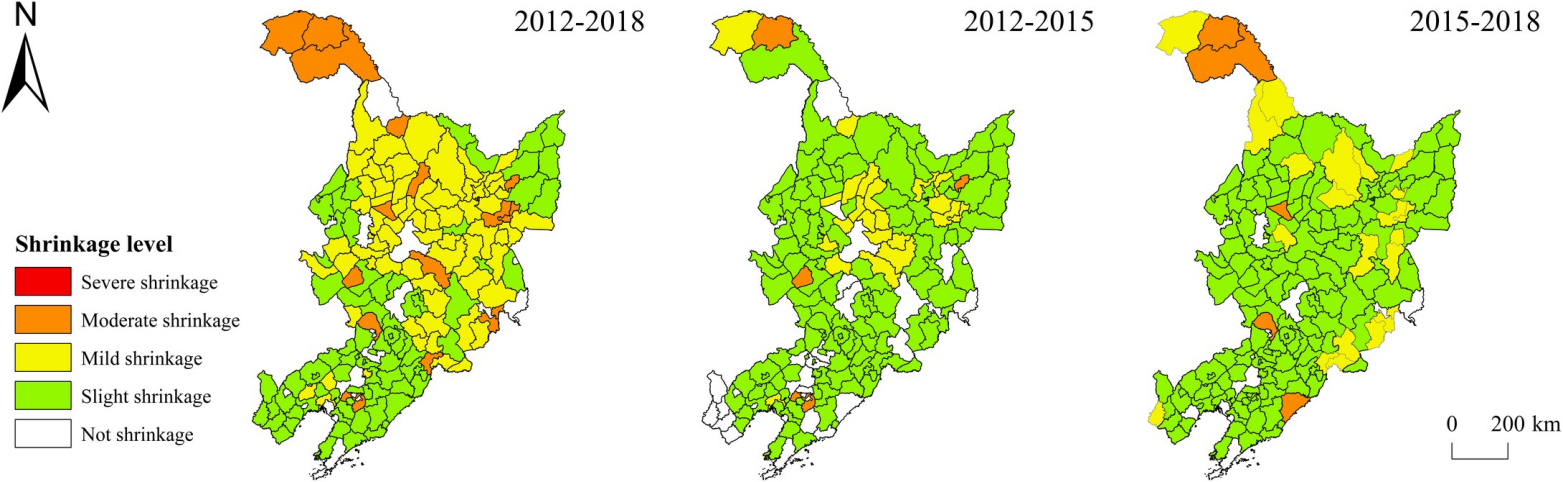
Spatial difference of population-related shrinkage of the three northeastern provinces. Note: The basic map came from a public map from the standard map service website of the Ministry of Natural Resources of China. The drawing approval number is GS (2016)1593.

In terms of the spatiotemporal difference of the population-related shrinkage in the provinces (Fig 3), the overall population-related shrinkage from 2012 to 2018 showed a trend of mild shrinkage in the north and slight shrinkage in the south, and a higher degree of shrinkage in the north than in the south. The moderately shrinking regions were scattered between the mildly and slightly shrinking regions in the form of dots. Regions without population-related shrinkage were mainly concentrated in the Harbin-Dalian Economic Zone. Neither the capital cities of the three provinces nor the municipal district of Dalian City showed population-related shrinkage, indicating that the central city maintained a strong appeal to the population under the overall background of regional population loss. Heilongjiang Province demonstrated the most severe shrinkage, and many municipal districts also shrank, especially a wide range of moderately shrinking regions in the surrounding area of Harbin City, which was not unrelated to the siphonic effect of the core city. From 2012 to 2015, the three provinces showed slight shrinkage, and the mildly shrinking regions were mainly distributed in the surrounding area of Harbin city. The 4 moderately shrinking regions were mainly distributed in dots, and moderate and mild shrinkage mainly appeared in county-level cities, municipal counties, and autonomous counties, while municipal districts shrank slightly. From 2015 to 2018, the scope of population-related shrinkage in the three provinces expanded, and some municipal districts in Heilongjiang Province experienced population-related shrinkage.

**Fig 4:**
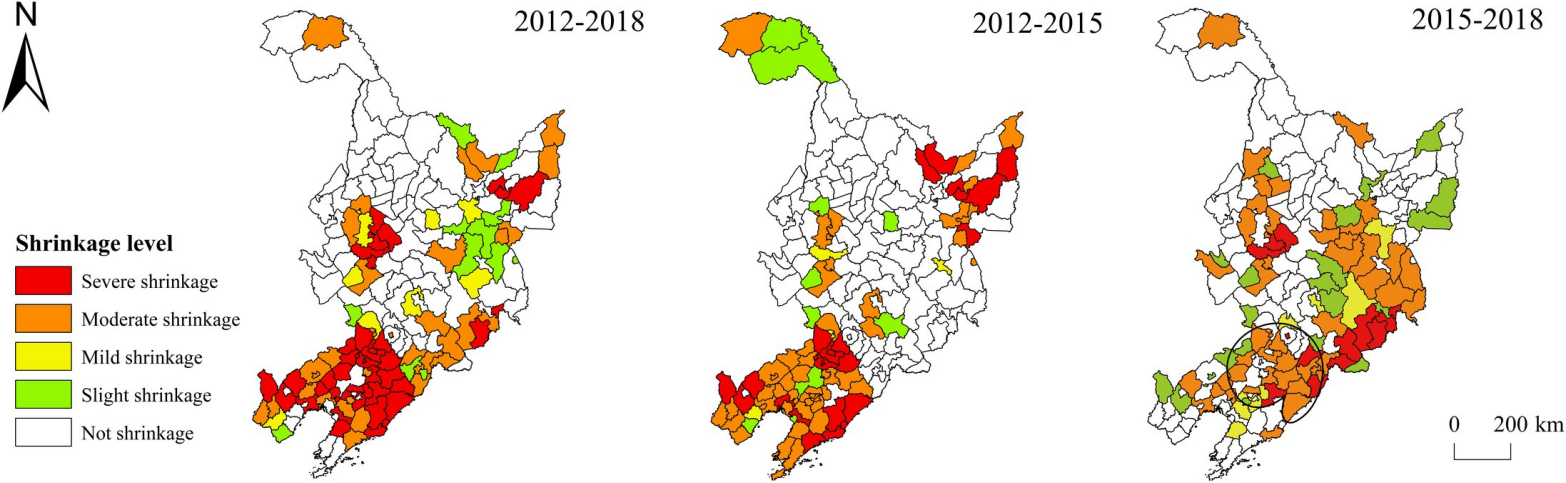
Spatial difference of economy-related shrinkage of the three northeastern provinces. Note: The basic map came from a public map from the standard map service website of the Ministry of Natural Resources of China. The drawing approval number is GS (2016)1593.

Although the scope of economy-related shrinkage was not as wide as population-related shrinkage, its degree was more serious (Fig 4), demonstrating that the population loss of the three provinces was accompanied by a relatively narrow but severe economic recession. The economically shrinking regions in the three provinces from 2012 to 2018 were mainly distributed at Liaoning Province, eastern Jilin Province, eastern Heilongjiang Province, and the junction between Heilongjiang and Jilin provinces, among which severely and moderately shrinking regions accounted for more than 40%. The most extensive and severe economy-related shrinkage was found in Liaoning Province. 44 out of the 55 regions were shrinking, and there were as many as 40 severely shrinking regions. However, most of the municipal districts did not sustain economy-related shrinkage. The overall economic development was characterized by “growth at the center and shrinkage at the periphery, indicating that most municipal districts in Liaoning Province still enjoyed considerable economic growth capacity, but the economic development of counties and cities other than municipal districts was facing difficulties. At the junction between Heilongjiang and Jilin provinces, there formed a shrinking region with the municipal district of Daqing City as the center, and shrinkage from the center to the periphery; there was a strip-shaped shrinking region from south to north in Heilongjiang Province. From 2012 to 2015, 52 of the 55 regions in Liaoning Province underwent economy-related shrinkage, of which 17 shrank severely and 30 shrank moderately; from 2015 to 2018, the scope and degree of economy-related shrinkage in Liaoning Province decreased remarkably, and the area around the municipal district of Shenyang City shrank moderately, forming a “q-shaped” shrinking region; the largest shrinking region during this period was found at the junction between Jilin and Heilongjiang provinces, mainly concentrated in Jilin City, Yanbian Prefecture, Mudanjiang City, and other cities; the municipal district of Daqing City did not shrink, but its peripheral area still sustained shrinkage.

**Fig 5:**
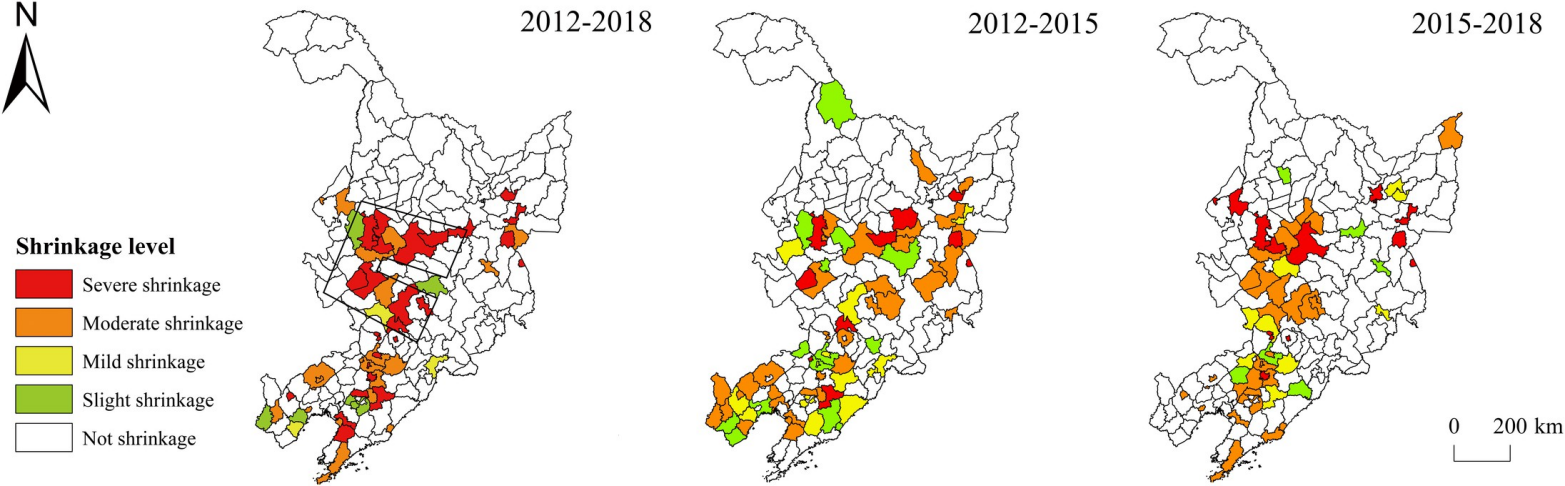
Spatial difference of space-related shrinkage of the three northeastern provinces. Note: The basic map came from a public map from the standard map service website of the Ministry of Natural Resources of China. The drawing approval number is GS (2016)1593.

Due to the rigidity of spatial expansion, the scope and extent of space-related shrinkage in the three provinces were not as great as population- and economy-related shrinkage (Fig 5). From 2012 to 2018, there were mainly two spatially shrinking regions in the three provinces: the “barbell-shaped” area at the junction between Heilongjiang and Jilin provinces and the northern area of Liaoning Province. The “barbell-shaped” area mainly included Changchun, Shuangliao, Songyuan, and other cities; the northern area of Liaoning Province mainly included Liaoyang, Benxi, Fushun, and other cities. From 2012 to 2015, the number of spatially shrinking regions accounted for more than 40%, which were distributed in dispersion on the whole. The relatively concentrated areas were mainly in the central and northwestern parts of Liaoning Province and southern Heilongjiang Province; between 2015 and 2018, the number of shrinking regions decreased, which were mainly distributed at the junction between Heilongjiang and Jilin provinces and northern Liaoning Province, consistent with the shrinking regions from 2012 to 2018.

**Fig 6:**
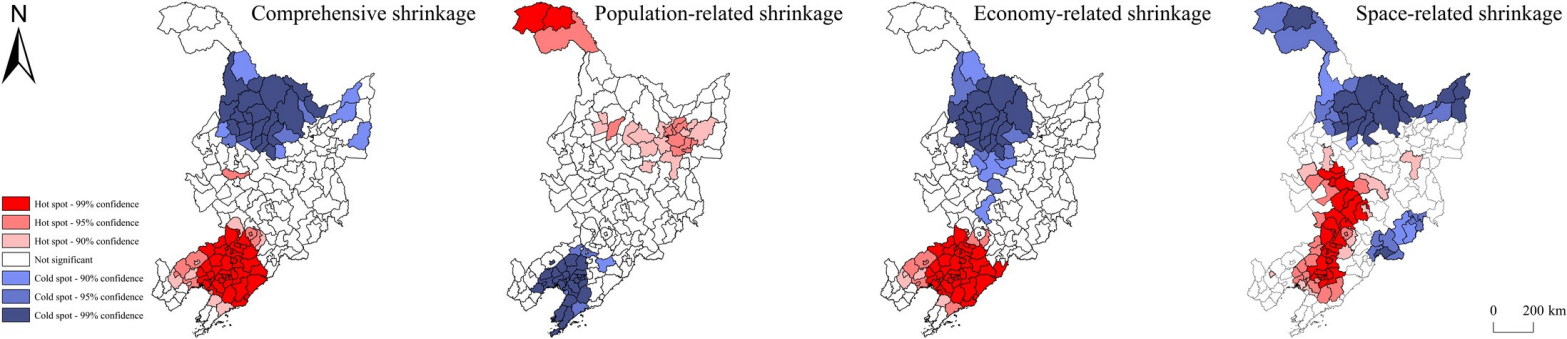
Cold-hot spots of regional shrinkage of the three northeastern provinces from 2012 to 2018. Note: The basic map came from a public map from the standard map service website of the Ministry of Natural Resources of China. The drawing approval number is GS (2016)1593.

The space-related shrinkage of the three provinces showed spatial centralization to some extent. *Moran’s I* for comprehensive, population-, economy-, and space-related shrinkage were 0.4917, 0.1282, 0.4342, and 0.4237, respectively, which all passed the significance test of 0.01. This suggested that the shrinking regions of the three provinces were not randomly distributed, but had a spatial correlation to some degree. Judging from the hotspot analysis (Fig 6), the hotspots of comprehensive shrinkage were mainly distributed in Liaoning Province; the hotspots of population-related shrinkage were distributed in the central and northwestern marginal areas of Heilongjiang Province; the hotspots of economy-related shrinkage were distributed in Liaoning Province; and the hotspots of space-related shrinkage were distributed in Jilin Province and the central part of Liaoning Province. In terms of the spatial difference and hotspot analysis of the regional shrinkage of the three provinces, Liaoning Province, which enjoys the highest level of economic development, experienced a relatively severe economy-related shrinkage, but the population-related shrinkage was the slowest. The provincial capital city Shenyang and the economic hub Dalian did not tend to shrink in a long period of time.

### The driving mechanism of regional shrinkage in the three provinces

The regional shrinkage in China is both similar and unique compared with western countries. The appearance and development of shrinkage are the result of a dominant factor or a combination of multiple factors [42]. The selection of influencing factors must take into consideration the spatiotemporal difference of regional shrinkage and the actual development in the three provinces. The heavy industry in the three provinces accounts for a large proportion, and industry remained an important pillar of their economic growth. The rise and fall of industrial enterprises largely affect their economic development. The traditional economic development model that relies dominantly on resource exploitation in the three northeastern provinces is unsustainable and must resort to other driving forces for long-term economic growth, such as the tertiary industry. Therefore, the industrial structure is also an important factor affecting the shrinkage of these provinces. According to the Petty-Clark Theorem, with the development of the economy, the share of the national income from the primary and secondary industries gradually decreases, while that from the tertiary industry tends to increase. Specifically, traditional resource-based industries still play a role in promoting the economy, but their excessive development deteriorates the living environment and thus forces the population to flow out. Worse still, problems such as low economic efficiency arise from the transition from labor-intensive industries to technology capital-intensive industries, which undermine residents’ income and urban economic development. This “pushing force”, together with the “pulling force” of economically developed areas, cause the population to leave their original places of residence, resulting in large-scale unemployment and population outflow. Agriculture serves as the foundation of the national economy and the core industry of the rural economy. The deterioration of agricultural production conditions or the improvement of production efficiency also leads to regional population loss and shrinkage. The population aging and low birth rate in the three northeastern provinces are becoming more and more grievous, and the changes in the population structure have led to the natural decrease of the regional population and the lack of labor force required for economic development. The three provinces enjoy a high share in the state-owned economy, and their economic development is highly dependent on the government. On the other hand, the natural conditions and the living standards of residents also affect the population flow. Therefore, while ensuring the data source, the regional shrinkage index was used as the dependent variable to analyze the influencing factors of regional shrinkage in light of natural conditions, industrial structure, agricultural development, industrial development, population structure, local finance, and living standards. On this basis, an indicator system for the influencing factors of regional shrinkage in the three provinces was constructed (Table 5). Among the indicators, the altitude reflects natural conditions, and the proportion of the secondary and tertiary industries represents changes in the industrial structure; the output value of agriculture, forestry, animal husbandry and fishery reflects the scale and achievement of agricultural production, whose change rate indicates agricultural development; industrial enterprises above the designated size are an important support for the development of regional industry and economy, whose change rate reflects industrial development; the change rate of population aging stands for the population structure; general public budget expenditure indicates the support of local finance to the people’s wellbeing, and its change rate is used to reflect local finance; the balance of resident deposits is an important manifestation of income, whose change rate reflects the living standards of residents.

**Table 5:** Influencing factor system of regional shrinkage of the three northeastern provinces.

Target layer	Criterion layer	Index layer	Symbol	VIF
Dependent variable	Shrinkage level	Regional shrinkage index	*Shrink*	-
Influencing factors	Natural conditions	Altitude	*Alt*	1.227
	Industrial structure	The proportion of the secondary industry	*Sec*	1.517
		The proportion of the tertiary industry	*Ter*	1.33
	Agricultural development	The output value of agriculture, forestry, animal husbandry and fishery	*Agr*	1.064
	Industrial development	The number of industrial enterprises above designated size	*Ind*	1.193
	Population structure	Population aging	*Agi*	1.071
	Local finance	Fiscal expenditure	*Fin*	1.129
	Living standards	The balance of resident deposits	*Res*	1.026

#### Regression results based on OLS

OLS was used to reveal the influence of various factors on regional shrinkage. The specific regression model was:

$$\mathrm{Shrink}=\beta_{1}\mathrm{Alt}+\beta_{2}\mathrm{Sec}+\beta_{3}\mathrm{Ter}+\beta_{4}\mathrm{Agr}+\beta_{5}\mathrm{Ind}+\beta_{6}\mathrm{Agi}+\beta_{7}\mathrm{Fin}+\beta_{8}\mathrm{Res}+\alpha$$

To avoid multicollinearity among multiple independent variables, a variance inflation factor (VIF) test was performed on them. The results revealed that the VIF value of each dependent variable was less than 5 (Table 5), suggesting that there was no obvious multicollinearity between the dependent variables. Stata was used to analyze the influencing factors of the regional contraction index from 2012 to 2018 based on OLS (Table 6). To enhance the reliability of the research results, the 8 independent variables were respectively subjected to regression analysis with the dependent variables (models 1–8), and then the 6 influencing factors together were taken as an independent variable for regression analysis with the dependent variables (model 9). From the regression results of individual influencing factors (models 1–8), the influence on the shrinkage index in descending order was: population aging, the proportion of the secondary industry, the share of the tertiary industry, the number of industrial enterprises above the designated size, fiscal expenditure, the output value of agriculture, forestry, animal husbandry and fishery, the balance of resident deposits, and the altitude. In Model 9, the regression coefficient of the proportion of the tertiary industry was the largest, followed by the proportion of the secondary industry, showing that the industrial structure had a significant impact on the shrinkage index. The regression coefficient of fiscal expenditure was greater than industrial enterprises above the designated size. The ranking of other factors regarding their impact on the shrinkage index did not change. The regression coefficients of the proportion of the secondary industry, the output value of agriculture, forestry, animal husbandry and fishery, the number of industrial enterprises above the designated size, fiscal expenditure, and the balance of resident deposits were all positive in the two regression model tests, showing a significant positive correlation. This revealed that the decline in the industrial economy, agricultural output, financial input from the local government and residents’ living standards increased the degree of regional shrinkage to a certain extent. The regression coefficients of the proportion of the tertiary industries were all negative in the two regression model tests, which may be related to the regional economic transformation. While the production efficiency in the process of industrial structure upgrading in some regions increased, the dependence on labor was weakened, which may cause population loss. The regression coefficients of population aging were negative in the two regression model tests. The increase of the elderly population meant the gradual disappearance of the demographic dividend and the reduction of the supply of labor resources, which increased the burden on social support and finance, resulting in the loss of labor force and constraints of economic development. The altitude and the balance of resident deposits failed the significance test in Model 9, showing that natural conditions and residents’ living standards had a certain impact on the shrinkage index, but the effects of the two were not significant under the combined effect of multiple factors.

**Table 6:** OLS regression of factors affecting regional shrinkage of the three northeastern provinces.

	Model 1	Model 2	Model 3	Model 4	Model 5	Model 6	Model 7	Model 8	Model 9
α	0.137	0.185	0.186	0.082	0.095	-0.767	-0.006	-0.033	-0.120
*Alt*	-0.013*** (-2.846)								-0.008 (-0.193)
*Sec*		0.383*** (6.412)							0.144** (2.092)
*Ter*			-0.243*** (-4.963)						-0.154*** (-3.073)
*Agr*				-0.045*** (-3.354)					-0.029** (-2.491)
*Ind*					0.137*** (4.906)				0.048* (1.702)
*Agi*						0.423*** (3.513)			0.112 (0.960)
*Fin*							0.134*** (5.813)		0.091*** (3.918)
*Res*								0.115*** (2.920)	0.038 (1.089)

Notes

* denotes statistical significance at the confident level of 0.1

** denotes statistical significance at the confident level of 0.05

*** denotes statistical significance at the confident level of 0.01. T statistics is in parentheses.

#### Regression results based on GWR

**Fig 7:**
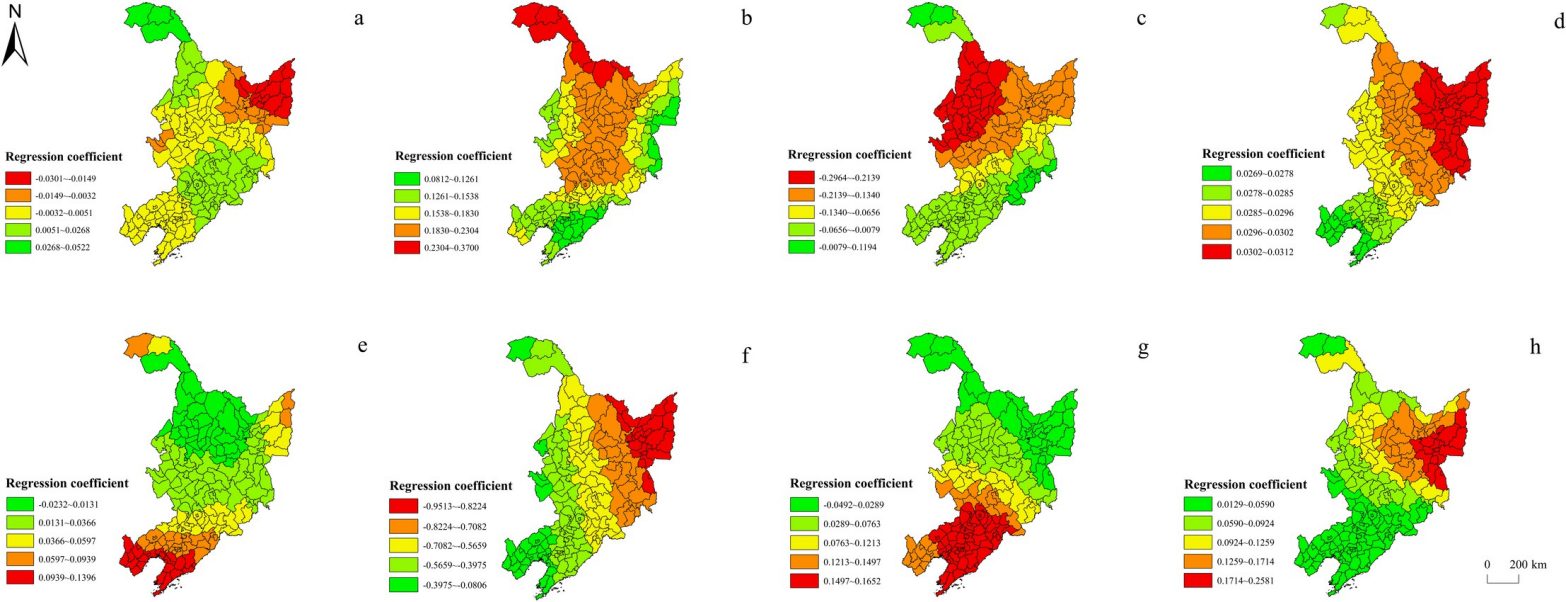
Spatial distribution of the regression coefficients of influencing factors based on GWR. a. altitude; b. the proportion of the secondary industry; c. the proportion of the tertiary industry; d. the output value of agriculture, forestry, animal husbandry and fishery; e. the number of industrial enterprises above the designated size; f. population aging; g. fiscal expenditure; h. the balance of resident deposits. Note: The basic map came from a public map from the standard map service website of the Ministry of Natural Resources of China. The drawing approval number is GS (2016)1593.

Compared with OLS, GWR can reveal more profoundly the spatial difference of factors affecting regional shrinkage [43]. Therefore, on the basis of OLS, GWR was used to analyze the intensity of different factors in different regions (Fig 7). Both GWR and OLS produced basically the same regression results in terms of the positive and negative correlations between various factors and the shrinkage index, but different factors showed different influences in different regions. The high values of the altitude regression coefficients appeared in the marginal areas such as the northeast and northwest of Heilongjiang Province, which were consistent with the hotpots of population-related shrinkage. These areas have a low level of economic development and the population tends to migrate as affected by natural conditions, hence shrinkage. The high values of the regression coefficient of the proportion of the secondary production were concentrated in the northwest and central area, and the maximum value occurred in Tahe County, Heilongjiang Province. The regression coefficient of the proportion of the tertiary industry gradually increased from southeast to northwest, and the minimum value appeared in Gannan County, Heilongjiang Province. The spatial distribution pattern of the regression coefficients of proportion of the tertiary industry was roughly similar and consistent with the pattern of population-related shrinkage, demonstrating the different attractiveness of different industries to the population, indicating that the three provinces should pay attention to further optimizing the industrial structure and enhancing the vitality of economic development when dealing with regional shrinkage, and attracting local and foreign labor in developing the tertiary industry. The regression coefficient of the output value of agriculture, forestry, animal husbandry and fishery also showed a decreasing trend from northeast to southwest, and the high-value center was located in the northeast of Heilong Province, which may be attributed to the important position of agriculture in Heilongjiang Province. Its total output value reached 562.43 billion yuan, ranking second in China. This indicated that Heilongjiang Province should pay attention to maintaining the basic position of agriculture in its development, and meanwhile be alert to the deterioration of agricultural production conditions leading to population loss. The regression coefficient of the number of industrial enterprises above the designated size showed a decreasing trend from the north and south to the center, and the high-value center was distributed in central-southern Liaoning and the northeast of Heilongjiang Province, which was consistent with the pattern of economy-related shrinkage, indicating that the industry remained an important pillar supporting the economic development of the three provinces, and economy-related shrinkage was even more serious in places such as central and southern Liaoning which are facing the challenges of transformation. The regression coefficient of population aging showed a decreasing trend from northeast to southwest, with the high-value center being distributed in the northeast of Heilongjiang Province where population shrinkage was the most serious, similar to the pattern of population-related shrinkage. According to the Seventh National Population Census, the proportion of the 60-year-old and 65-year-old and above permanent residents in Heilongjiang Province registered 23.22% and 15.61%, respectively, with its aging degree ranking third in China. In fact, population aging and regional shrinkage are mutually causal. On the one hand, the loss of young and middle-aged labor force in the process of regional shrinkage leads to an aging population structure. On the other hand, the elderly population has limited impetus for economic development, which is not conducive to the development of commercial services. As a result, it is difficult for the region to attract young adults and lead to their loss. The regression coefficients of fiscal expenditure showed a downward trend from south to north, which was similar to the pattern of economy-related shrinkage. Liaoning Province, which had the most severe economy-related shrinkage, had the largest regression coefficient. The growth rate of its fiscal expenditure (17.00%) from 2012 to 2018 was much lower than the average level of the whole country (75.39%), suggesting that insufficient financial support from the government was also an important reason for economy-related shrinkage [44]. The regression coefficients of the balance of resident deposits showed a downward trend from northeast to southwest, and the maximum value appeared in Hulin City, Heilongjiang Province, which was basically consistent with the pattern of population-related shrinkage, especially northeast Heilongjiang Province, where the regression coefficient was the highest, seen severe population-related shrinkage.

#### Influencing factors of regional shrinkage

Regression analysis was performed on population-related, economy-related and space-related shrinkage to explore whether there is any difference in their influencing factors. Their OLS equations were as follows:

$${\mathrm{Shrink}}_{p}=‐0.009\mathrm{Alt}+0.186\mathrm{Sec}-0.062\mathrm{Ter}-0.218\mathrm{Agi}-0.012\mathrm{Agr}+0.023\mathrm{Ind}+0.058\mathrm{Fin}+0.154\mathrm{Res}-0.034$$

$${\mathrm{Shrink}}_{e}=‐0.010\mathrm{Alt}+0.355\mathrm{Sec}-0.272\mathrm{Ter}-0.341\mathrm{Agi}-0.032\mathrm{Agr}+0.119\mathrm{Ind}+0.109\mathrm{Fin}+0.009\mathrm{Res}-0.432$$

$${\mathrm{Shrink}}_{s}=0.001\mathrm{Alt}+0.279\mathrm{Sec}-0.265\mathrm{Ter}-0.206\mathrm{Agi}-0.023\mathrm{Agr}+0.032\mathrm{Ind}+0.180\mathrm{Fin}+0.153\mathrm{Res}+0.013$$

Population aging, the proportion of the secondary industry and the balance of resident deposits exerted the greatest impact on population-related shrinkage. Population aging increases the burden on social support among the working population and lead to population outflow; meanwhile, when the proportion of the elderly population is too high, there is a potential risk of population decline. The secondary industry was once the backbone of the development of the three northeastern provinces in the early stage. With the exhaustion of resources, a single resource-based industry could not provide more employment opportunities, thus resulting in population outflow. According to the Push-Pull Theory, another important reason for population shrinkage is that the local income level is lower, and the population flows to economically developed areas for better living conditions.

The proportion of the secondary industry, population aging, and the proportion of the tertiary industry were the three most important factors affecting economy-related shrinkage. The regression coefficient of the proportion of the secondary industry was the largest and positive, indicating its pivotal role on the economic development of the three northeastern provinces. Population is an important driving force for economic development. The aggravation of population aging has led to a relative decline in the working-age population and reduced labor efficiency, thus hindering economic development. The regression coefficient of the tertiary industry was negative, suggesting that amidst the transition from the secondary to the tertiary industry, there may be a “pain period” characterized by low economic benefits. This further indicated that the three northeastern provinces should not work towards deindustrialization blindly; rather, they should accelerate the competitiveness of the tertiary industry.

In terms of space-related shrinkage, the proportion of the secondary industry, the proportion of the tertiary industry, and population aging saw the largest regression coefficients. The decline of the secondary industry in the old industrial base in Northeast China resulted in a large number of abandoned and idle industrial land, forming a decayed area and causing space-related shrinkage. The underdeveloped tertiary industries such as commercial services, tourism, and real estate led to unsteady macroeconomy, contributing to vacant land and buildings and finally space-related shrinkage. The impact of aging on space-related shrinkage was mainly manifested in the reduced economic vitality at night and vacant buildings due to the slow life of the elderly population, limited spending power, and low desire to buy houses. Moreover, fiscal expenditure showed a relatively large regression coefficient, indicating that the government’s financial crisis that led to the collapse of infrastructure also represented an important factor for space-related shrinkage.

## Discussion

### The relationship between population-, economy- and space-related shrinkage

**Fig 8:**
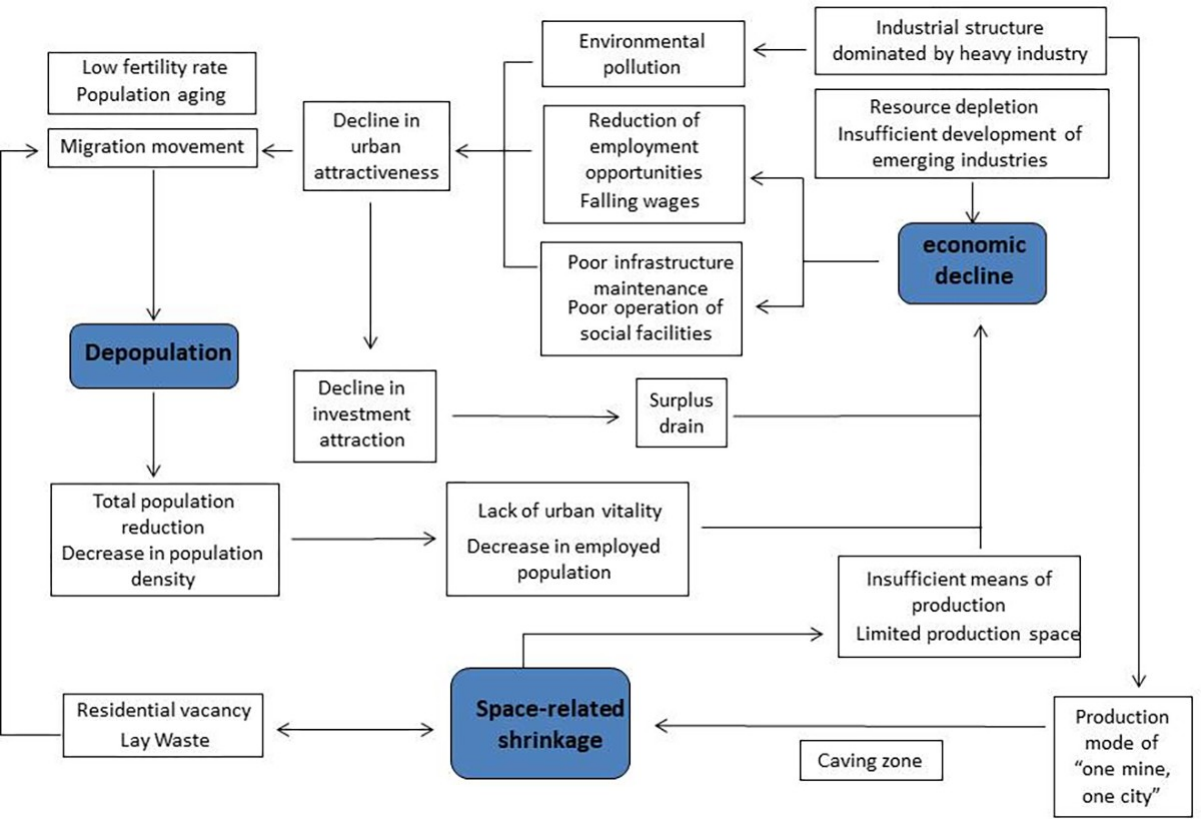
Driving mechanism of regional shrinkage of the three provinces in the northeast of China.

The influencing factors and actual conditions of the regional shrinkage in the three provinces were synthesized to further discuss its driving mechanism in depth (Fig 8). The three dimensions of population, economy, and space interact with and influence each other. The shrinkage of one dimension may affect other dimensions, and vice versa, resulting in a vicious circle of shrinkage. Firstly, the natural population growth rate directly affects the changes in population size. For a long time, the total fertility rate of the three provinces has been ranked at the bottom of the country, which is significantly lower than the replacement level. The provinces have fallen into a “low fertility trap”, which has resulted in a series of population problems such as population loss, labor shortage, and aging [16, 45]. The working-age population had to bear the rising dependency coefficient while the number itself kept declining, and the social burden continued to increase [46]. Under the combined effect of these factors, the labor population gradually emigrated, while the elderly population emigrated in a small number, which led to a vicious circle of the above-mentioned problems, which in turn resulted in problems such as reduced social vitality and restricted economic development.

Secondly, the industrial shrinkage caused by resource exhaustion and the industrial structure dominated by heavy industries severely hampered economic development, coupled with the insufficient development of emerging industries, resulting in an economic recession [47]. The old industrial base in the northeast part of China used to develop and grow with rich mineral resource reserves and a strong heavy industry foundation. However, with the exhaustion of resources and the inefficiency and pollution of low-end industries, as well as the inability to innovate and transform, the traditional heavy industry-based production pattern was facing the dilemma of overcapacity as well as short and low-end industrial chain. Obviously, it was unable to meet the needs of industrial transformation and upgrading nowadays. In addition, the development of emerging industries was lagging behind and could not provide sufficient jobs. The proportion of state-owned enterprises and state-owned economy in many regions was high, and reform was difficult for this reason. The exhaustion of resources combined with a single industrial structure has led to a lack of vitality in the regional economic development, followed by industrial decline and population loss.

Finally, the living environment such as wage level, employment opportunities, urban environment, supporting infrastructure, etc. directly affects residents’ willingness to live in a certain area [48]. Problems such as declining wages of residents, reduced employment opportunities, deteriorating urban environment, and imperfect supporting infrastructure will directly reduce the attractiveness of the city to residents. Together with the improvement of traffic conditions, this will accelerate population loss. The long-term single industrial structure has caused irreparable damage to the regional environment, such as the subsidence area of coal resource-based cities and the air quality problems of Anshan due to steel production. The wages of employees in the three provinces were lower than the national average, and there was a lack of attraction for the employed population, thereby resulting in population loss. The decrease in the total population led to insufficient employment, which in turn hindered economic development; meanwhile, the decrease in population and the decrease in density resulted in the vacancy of residential houses and caused space-related shrinkage [49].

### Different mechanisms of regional shrinkage between China and other countries

Regional shrinkage is the result of multiple factors, whether in China or in other countries. These factors include globalization, deindustrialization, post-socialist transformation, and the global financial crisis [33]. However, the driving mechanism of regional shrinkage differ due to the different political, economic, geographical and social backgrounds, especially the difference in the urbanization process between China and western countries. At present, China is in the middle stage of urbanization whereby the population migrates from the countryside to the cities, leading to considerable regional shrinkage especially in countries and small towns in China. This shrinking pattern is spatially manifested as “expansion in the core and shrinkage at the edge”, as is the case around Shenyang municipal districts. In contrast, some western countries have entered the late stage of urbanization, and the phenomenon of suburbanization has begun to appear. Capital and population migrate from central areas to peripheral areas, resulting in largely deserted central cities, lost population, and severe shrinkage in cities such as Chicago and Philadelphia [20]. However, the shrinkage of urban center does not mean that the entire region is shrinking. For example, although Detroit in the “Rust Belt” experiences a severe recession in the urban center and the first suburbs, but its overall population and economy continue to grow, showing a pattern of “shrinkage in the core and expansion at the edge” [50].

Globalization has changed the socio-spatial order and affected the geographical distribution of economic and political activities and the process of urbanization [51]. The globalization of the economy, capital and production process has led to the single industry-dominated regional shrinkage in western countries. The impact of globalization on China is manifested in the concentration of capital in developed regions. Due to the unbalanced regional economic development, the population flows from the western part to the eastern part of China, and this is the reason for the huge population loss in the three northeastern provinces. The economic transformation and population loss caused by deindustrialization were the main reasons for the shrinkage of old European and American industrial cities such as Liverpool and Detroit in the early days [52]. However, China, including the three northeastern provinces, has not experienced de-industrialization on the whole, excerpt for regional de-industrialization in Shanxi Province and other places, which results in the shrinkage of Lvliang. In the “Rust Belt” of the northeastern United States, racial prejudice also represents an important reason for the shrinkage of urban centers in Cleveland, Detroit, and Pittsburgh [53]. By contrast, this phenomenon does not appear in China. Aging and low birthrate are the leading factors of shrinkage in Germany and Japan [54]. Population aging is also a major challenge facing China, especially the three northeastern provinces. Now China has become an “aging” society. By the end of 2021, China’s population aged 60 years and above accounted for 18.70%, of which nearly 200 million were 65 years old and above. Moreover, the aging level of rural areas was significantly higher than that of urban areas, which indicates a higher risk of rural shrinkage than urban shrinkage. In addition to “passive shrinkage”, China’s cities featuring first-tier super-large scales and fragile resources and environment have begun to decrease and “shrink actively”. On the whole, the driving mechanism of China’s regional shrinkage is more diverse, and the shrinkage is intertwined with the growth brought about by urbanization and reindustrialization, thus resulting in fragmented shrinkage in some areas [55, 56].

## Conclusions and policy implications

### Conclusions

The regional shrinkage in the three provinces had a wide range but the degree lessened slightly. The comprehensive shrinkage was characterized by a wide range. Among them, the population-related shrinkage was the most extensive, the economy-related shrinkage was the most severe, and the space-related shrinkage was relatively mild. From the perspective of temporal evolution, the scope of comprehensive, economy-, and space-related shrinkage was reduced while the degree lessened, but the scope of population-related shrinkage expanded.

There was remarkable spatiotemporal difference between different dimensions of the regional shrinkage in the three provinces. Comprehensive shrinkage was mainly moderate, and there formed three major areas spatially, with the hotspots mainly distributed in Liaoning Province; economy-related shrinkage was the most serious in Liaoning Province, showing an economic development trend of “growth at the center and shrinkage at the periphery”; population-related shrinkage displayed a higher degree in the north than in the south, and the areas without population-related shrinkage were mainly concentrated in the Harbin-Dalian Economic Belt; there formed two large areas with space-related shrinkage, with the hotspots distributed in Jilin Province and central Liaoning Province. The space-related shrinkage of the three provinces showed spatial centralization to some extent.

The influencing factors of regional shrinkage were diverse and there were spatial differences. The proportion of the secondary industry had the greatest impact on the shrinkage index, and the areas with strong influence were concentrated in the northwest and central part; the proportion of the tertiary industry was negatively correlated with the shrinkage index, and the intensity of the influence gradually increased from the southeast to the northwest; the number of industrial enterprises above the designated size and fiscal expenditure were ranked third and fourth, respectively, and the influence intensity decreased from the north and south to the center and from south to north, respectively; the influence intensity of the balance of resident deposits decreased from northeast to southwest; the high values of the altitude regression coefficients appeared on the edge of Heilongjiang Province.

The driving mechanism of the regional shrinkage in the three provinces has its own characteristics. The three core dimensions of regional shrinkage interact with and influence each other. Low fertility, aging, and population mobility cause population decline, and inappropriate industrial structure and resource exhaustion lead to economic recession; insufficient employment caused by population decline in turn aggravates economic recession, and insufficient urban attractiveness caused by economic recession worsens population decline in turn. Population decline and economic recession trigger space-related shrinkage, which in turn aggravates population decline and economic recession.

### Policy implications

Like cities, regions have a life cycle of, formation, development and decline. Therefore, it is necessary to view regional shrinkage dialectically and recognize the irreversibility of some regional shrinkage, and properly deal with the opportunities and challenges brought about by shrinkage. It is especially necessary to pay attention to the shrinkage of core cities in regional development. It is advised to develop coping strategies according to the degree, pattern and driving mechanism of regional shrinkage as well as regional development characteristics and functional positioning based on local conditions.

Firstly, it is crucial to develop a development concept of shrewd shrinkage and reasonably control the regional scale. Excessive pursuit of growth and expansion under the traditional growthist model is not conducive to the sustainable development of shrinking regions. It is thus advised to learn from the experience of western countries in coping with shrinkage, adjust the traditional growth-oriented development strategy, and develop a development concept of shrewd shrinkage. It is important to optimize and reorganize regional population and economic factors in line with regional development trends, guide population and construction land quota in shrinking areas to reasonably concentrate in growth areas, and curb disorderly spatial development patterns. It is necessary to guide the effective withdrawal of population, land and resources in shrinking villages, improve land use efficiency, and optimize and adjust the rural spatial structure. It is also essential to shrink the industrial and spatial scale of cities, develop tock land, and improve the urban living environment.

Secondly, it is crucial to implement a revival-oriented development strategy to enhance regional core competitiveness. Regional shrinkage is largely caused by industrial recession. For example, resource-based cities such as Anshan and Benxi have long suffered from resource depletion and a single industrial structure, resulting in stagnant and even retrogressive economic development, which in turn leads to population-related and space-related shrinkage. For regions suitable for vigorous development, it is necessary to formulate growth-oriented development strategies, transform and upgrade traditional industries with high-tech, promote the extension and integration of closely related industries, and optimize and upgrade the industrial structure, thereby forming advantageous industries and enhancing core competitiveness. For instance, Yichun City may integrate and develop tourism based on its abundant forest resources, and promote the construction of forest tourism bases and agricultural industrial parks.

Thirdly, it is essential to strengthen the government’s macro-control, optimize the regional configuration, give full play to the advantages of the Chinese government in the administration, land, taxation and other resources, and focus on coping with regional shrinkage. First, it is crucial to formulate a planning model suitable for the shrinking area, and promote the transformation and development of the shrinking area through planning measures such as the development or storage of abandoned land, the reuse of industrial heritage, and the improvement of the ecological environment. Then, it is necessary to create a flexible and retractable spatial structure to enhance the region’s ability to cope with various uncertainties, crises and challenges. Finally, it is important to strive for financial transfer payments to alleviate the dilemma of economic development with the support of national policies and pay attention to the reemployment of laid-off workers by increasing employment opportunities and reducing unemployment.

## Supporting information

S1 Data (XLSX)

S1 File Cover letter corrected. (DOCX)
