# Supplementary material for: Spatiotemporal evolution and driving mechanism of regional shrinkage at the county scale: The three provinces in northeastern China
Source: PLoS One. 2022 Aug 22;17(8):e0271909. doi: 10.1371/journal.pone.0271909 (PMC9394843; doi:10.1371/journal.pone.0271909)
Supplement: S1 File — (DOCX) [file pone.0271909.s002.docx]

Dear editor,

On behalf of all authors, I am delighted to submit a manuscript entitled ‘Spatiotemporal evolution and driving mechanism of regional shrinkage at the county scale: The three provinces in the northeast of China’ to your appreciation and to the editorial analysis of Plos One, as to the possibility of its publication.

Urban shrinkage has been witnessed often throughout the history of Western cities. The western context is difficult to explain China’s shrinkage. The research of regional shrinkage is of more practical significance, under China’s current administrative system. The three provinces in the northeast of China are the most severe and typical shrinking region in China. We want to explore the spatiotemporal evolution and driving mechanism of regional shrinkage of the three provinces in the northeast of China.

In this paper, we established an evaluation system of regional shrinkage from the three dimensions of population, economy and space. We used shrinkage model, subjective and objective weighting, Moran’s *I* to analyze spatiotemporal difference of regional shrinkage in the three provinces. And we used ordinary least squares and geographically weighted regression to analyze the driving mechanism of regional shrinkage in the three provinces. We believe this manuscript is valuable for all the researchers who are interested in human geography and urban geography.

The manuscript has not been published before and is not being considered for publication elsewhere. All authors have contributed to the creation of this manuscript for important intellectual content and read and approved the final manuscript. We declare there is no conflict of interest.

**We are sorry that there was an error in filling in “Financial Disclosure”. We have provided the correct information in the fund information section as required.** **The specific Financial Disclosure is as follows:**

**The study was supported by the National Social Science Foundation of China (No. 20BJY070). The funders play no role in the study design, data collection and analysis, decision to publish, or preparation of the manuscript.** **No authors received a salary from any of our funders.**

Many thanks for your time and consideration.

Yours Sincerely,

Prof. Wang

(On behalf of co-authors)

College of Geography and Environment, Shandong Normal University

No.1, Daxue Road, Changqing District, Jinan, Shandong Province, China

Tel: 13869192938	Email: 404122665@qq.com	Fax: 0531-86182550
